# Supplementary material for: Short report: Plasma based biomarkers detect radiation induced brain injury in cancer patients treated for brain metastasis: A pilot study
Source: PLoS One. 2023 Nov 28;18(11):e0285646. doi: 10.1371/journal.pone.0285646 (PMC10684068; doi:10.1371/journal.pone.0285646)
Supplement: S1 Fig — Individual patients`classification to RBI and non-RBI tumor status during follow-up. During follow-up patient was classified to radiation-induced brain injury (RBI) or non-RBI tumor status according to clinical and radiological criteria. Each patient was able to be classified to more than one group in different time point along the follow-up period. Patients with no available clinical and radiographic data for analysis were excluded. RBI: radiotherapy induced brain injury; ED: early delayed; LD: late delayed; TR: tumor response; PD: progressive disease; SD: stable disease; URP: undetermined radiological progression; E: excluded. (DOCX) [file pone.0285646.s001.docx]

**
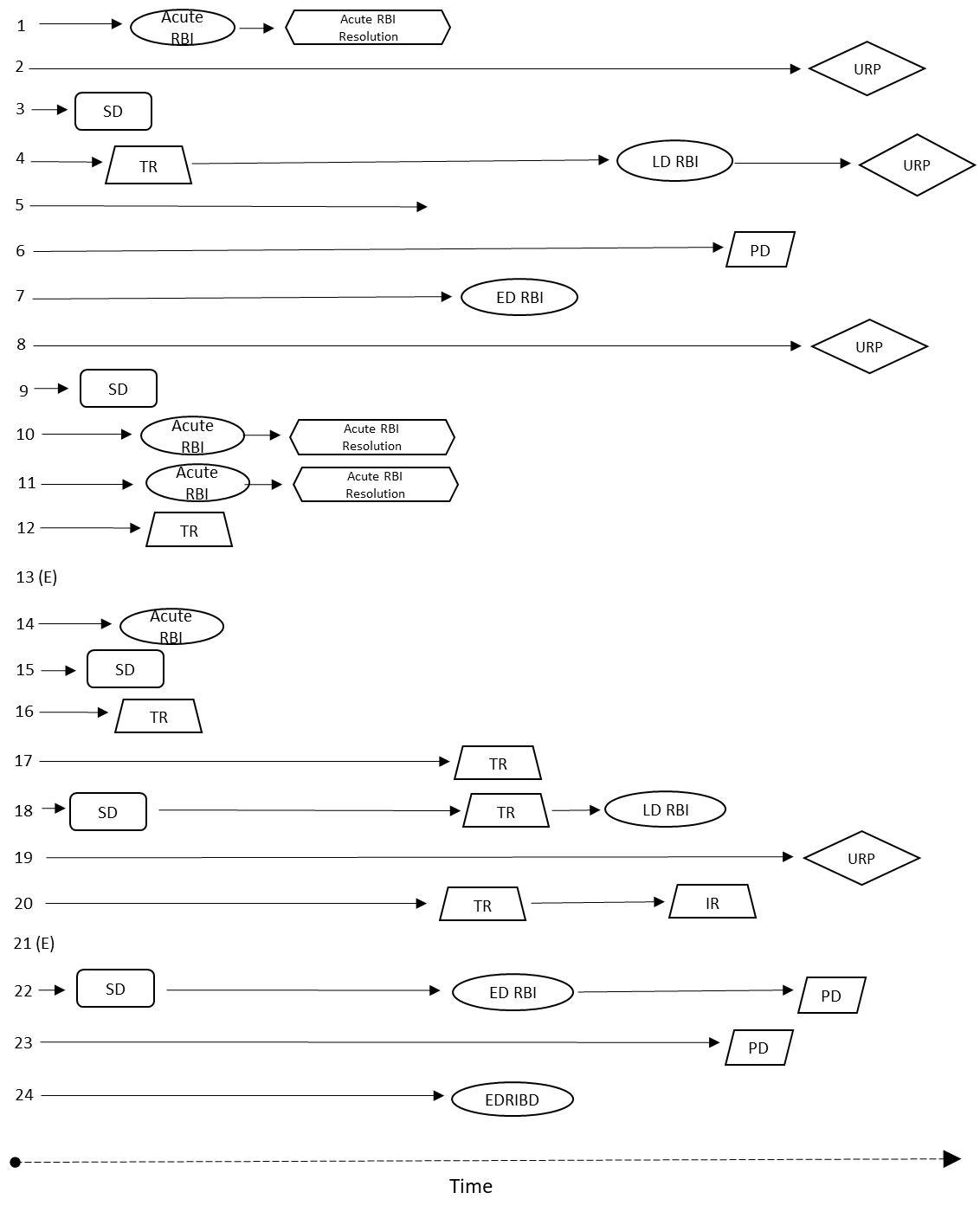
**

**Figure S1**: Individual patients` classification to RBI and non-RBI tumor status during follow-up. During follow-up patient was classified to radiation-induced brain injury (RBI) or non-RBI tumor status according to clinical and radiological criteria. Each patient was able to be classified to more than one group in different time point along the follow-up period. Patients with no available clinical and radiographic data for analysis were excluded. RBI: radiotherapy induced brain injury; ED: early delayed; LD: late delayed; TR: tumor response; PD: progressive disease; SD: stable disease; URP: undetermined radiological progression; E: excluded.
