# Supplementary material for: Short report: Plasma based biomarkers detect radiation induced brain injury in cancer patients treated for brain metastasis: A pilot study
Source: PLoS One. 2023 Nov 28;18(11):e0285646. doi: 10.1371/journal.pone.0285646 (PMC10684068; doi:10.1371/journal.pone.0285646)
Supplement: S3 Fig — BncfDNA levels (copies/ml) during the first 6 months after brain radiotherapy show significant increase in bncfDNA correlating to clinical and imaging manifestations of Early-Delayed RBI (marked by red arrowhead) in 3 Patients. Each graph represents a different patient. Each colored line represents a specific tissue origin of bncfDNA as detailed in the key (astrocytes, neurons, oligodendrocytes). Total bncfDNA marked in purple represent the mean summation of all 3 tissue types’ values. Mean baseline levels of bncfDNA among healthy individuals are: total bncfDNA (mean 1.32 copies/ml, std 3.2), astrocytes cfDNA (mean 1.76, std 5.4), oligodendrocytes cfDNA (mean 0.5, std 2.7), neurons cfDNA (mean 0.9, std 2.9). BncfDNA: brain-derived circulating DNA; RBI: radiation-induced brain Injury. (DOCX) [file pone.0285646.s003.docx]

Patient # 22 (SRS)

Patient # 7 (WBRT)


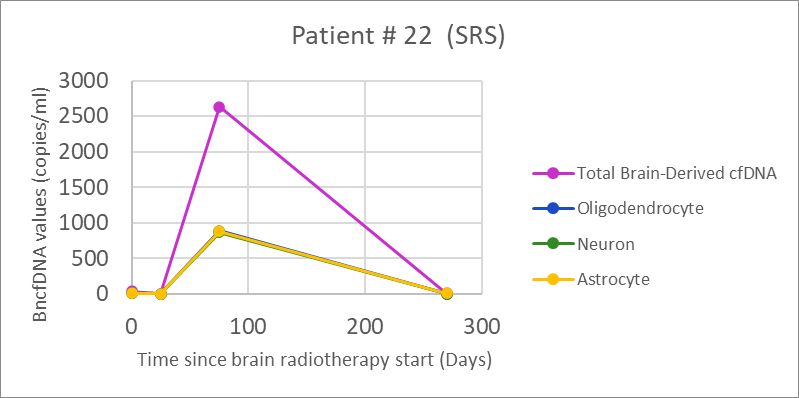

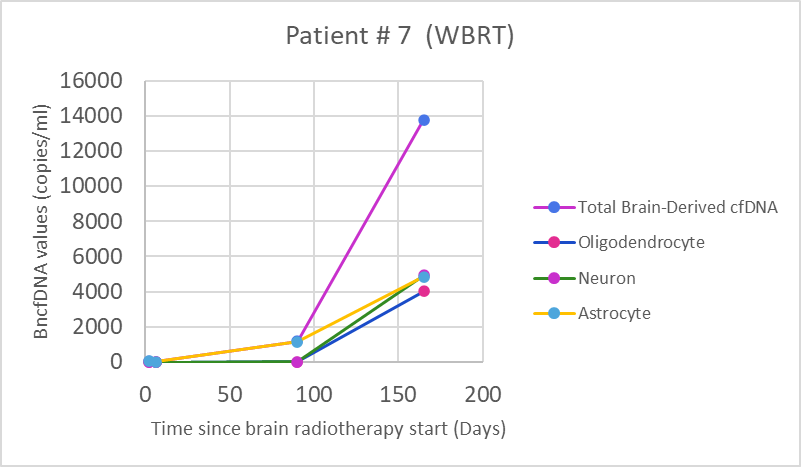


BncfDNA values (copies/ml)

BncfDNA values (copies/ml)

Time since brain radiotherapy start (Days)

Time since brain radiotherapy start (Days)

Patient # 24 (SRS)


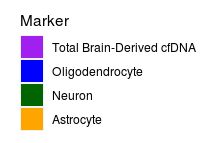


Total brain derived cfDNA

Oligodendrocyte derived cfDNA

Neuron derived cfDNA

Astrocyte derived cfDNA

RBI


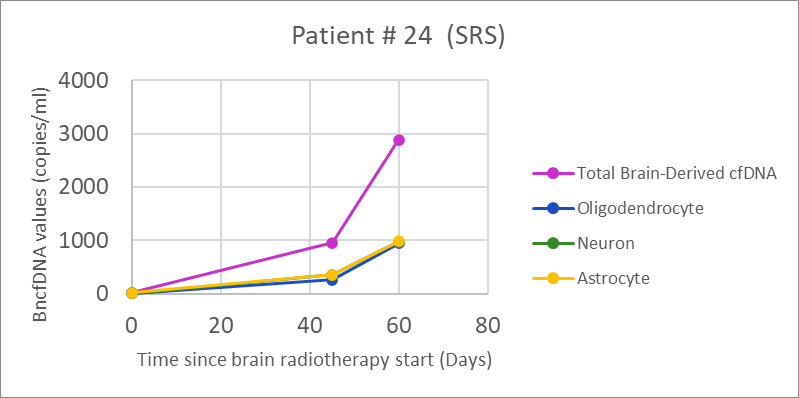


BncfDNA values (copies/ml)

Time since brain radiotherapy start (Days)
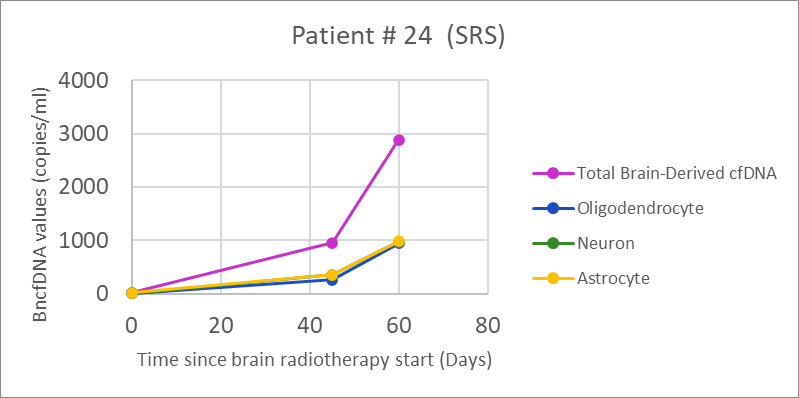


**Figure S3:** **BncfDNA levels in Early-Delayed RBI.** BncfDNA levels (copies/ml) during the first 6 months after brain radiotherapy show significant increase in bncfDNA correlating to clinical and imaging manifestations of Early-Delayed RBI (marked by red arrowhead) in 3 Patients. Each graph represents a different patient. Each colored line represents a specific tissue origin of bncfDNA as detailed in the key (astrocytes, neurons, oligodendrocytes). Total bncfDNA marked in purple represent the mean summation of all 3 tissue types’ values. Mean baseline levels of bncfDNA among healthy individuals are: total bncfDNA (mean 1.32 copies/ml, std 3.2), astrocytes cfDNA (mean 1.76, std 5.4), oligodendrocytes cfDNA (mean 0.5, std 2.7), neurons cfDNA (mean 0.9, std 2.9). BncfDNA: brain-derived circulating DNA; RBI: radiation-induced brain Injury.
