# Supplementary material for: Short report: Plasma based biomarkers detect radiation induced brain injury in cancer patients treated for brain metastasis: A pilot study
Source: PLoS One. 2023 Nov 28;18(11):e0285646. doi: 10.1371/journal.pone.0285646 (PMC10684068; doi:10.1371/journal.pone.0285646)
Supplement: S5 Fig — BncfDNA levels (copies/ml) during follow-up after brain radiotherapy in 7 patients with tumor response (TR), marked by blue arrow. Each graph represents a different patient. Each colored line represents a specific tissue origin of bncfDNA as detailed in the key (astrocytes, neurons, oligodendrocytes). Total bncfDNA marked in purple represent the mean summation of all 3 tissue types’ values. Mean baseline levels of bncfDNA among healthy individuals are: total bncfDNA (mean 1.32 copies/ml, std 3.2), astrocytes cfDNA (mean 1.76, std 5.4), oligodendrocytes cfDNA (mean 0.5, std 2.7), neurons cfDNA (mean 0.9, std 2.9). BncfDNA: brain-derived circulating DNA. (DOCX) [file pone.0285646.s005.docx]

Patient # 5 (WBRT)

Patient # 17 (SRS)


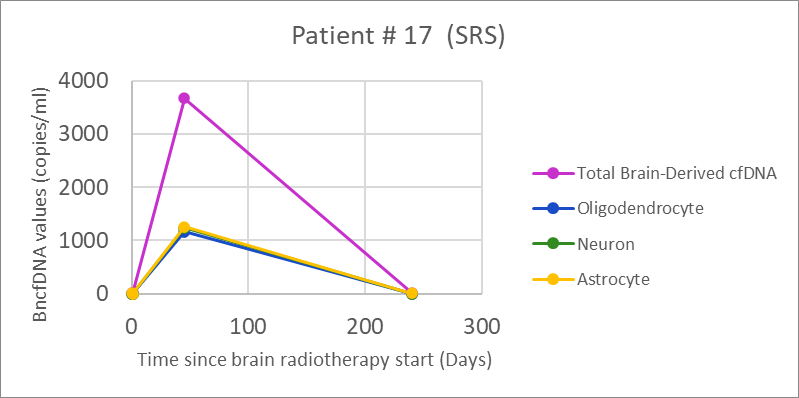

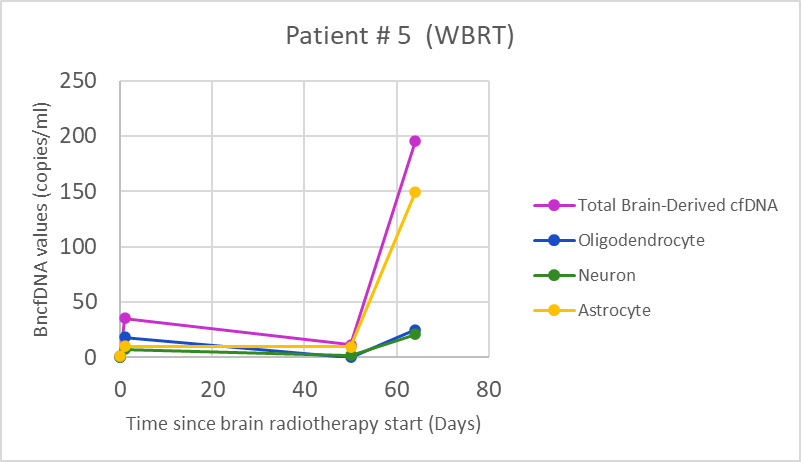


BncfDNA values (copies/ml)

BncfDNA values (copies/ml)

Time since brain radiotherapy start (Days)

Time since brain radiotherapy start (Days)

Patient # 20 (SRS)

Patient # 18 (SRS)


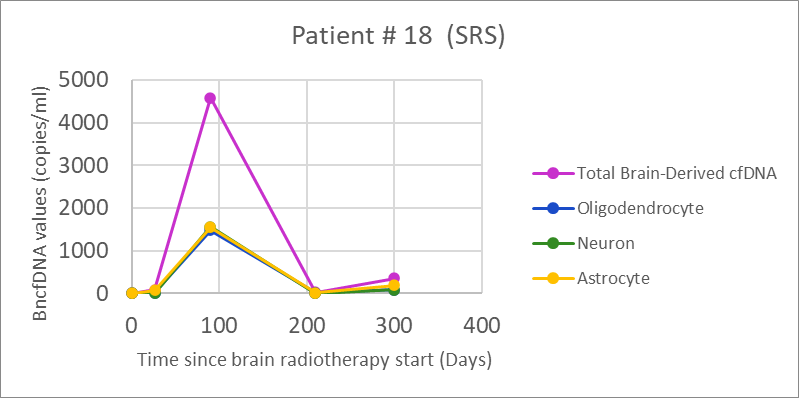

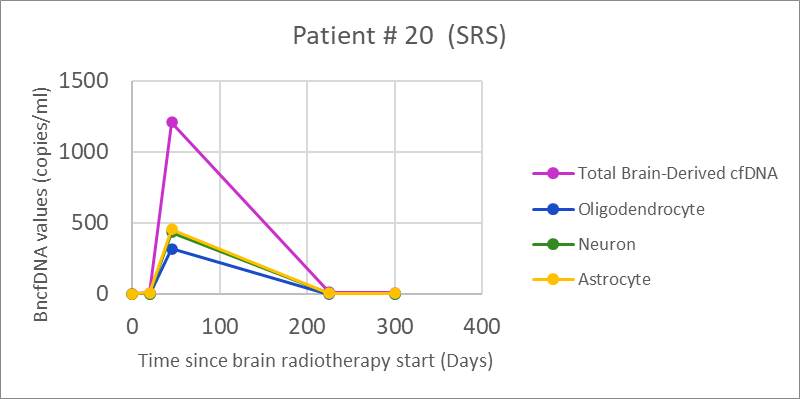


BncfDNA values (copies/ml)

BncfDNA values (copies/ml)

Time since brain radiotherapy start (Days)

Time since brain radiotherapy start (Days)


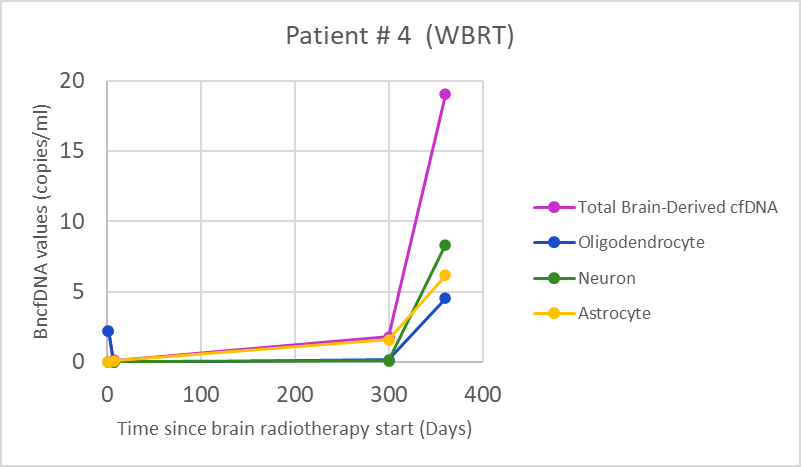


BncfDNA values (copies/ml)


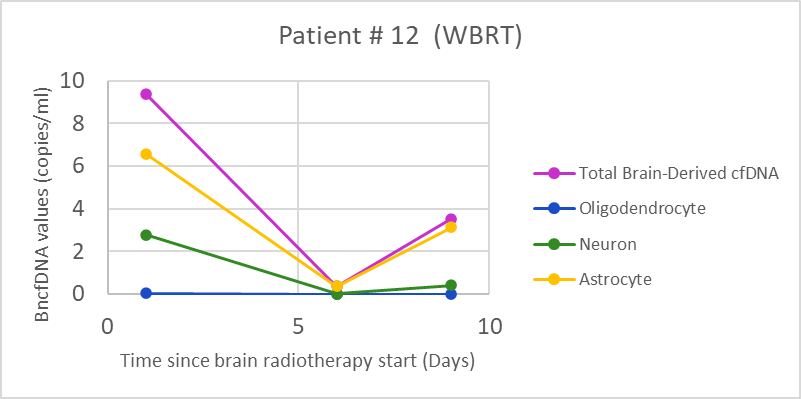


BncfDNA values (copies/ml)

Patient # 12 (WBRT)

Patient # 4 (WBRT)

Time since brain radiotherapy start (Days)

Time since brain radiotherapy start (Days)

Patient # 16 (WBRT)


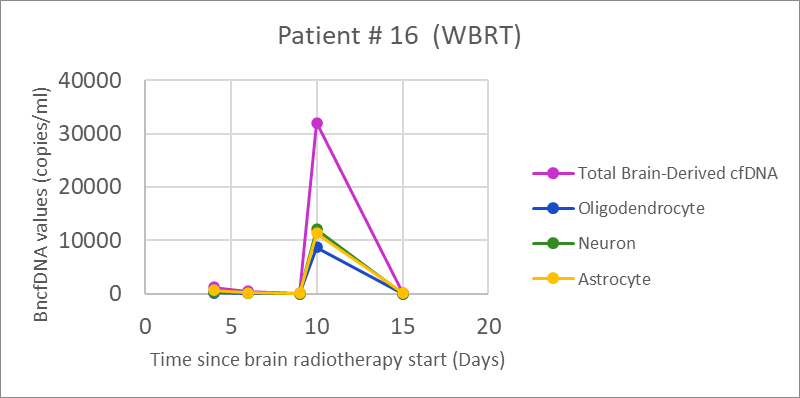


BncfDNA values (copies/ml)

Time since brain radiotherapy start (Days)


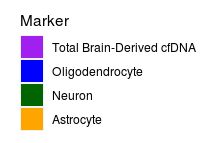


Total brain derived cfDNA

Oligodendrocyte derived cfDNA

Neuron derived cfDNA

Astrocyte derived cfDNA

TR

Time since brain radiotherapy start (Days)

**Figure S5**: **BncfDNA levels in TR.** BncfDNA levels (copies/ml) during follow-up after brain radiotherapy in 7 patients with tumor response (TR), marked by blue arrow. Each graph represents a different patient. Each colored line represents a specific tissue origin of bncfDNA as detailed in the key (astrocytes, neurons, oligodendrocytes). Total bncfDNA marked in purple represent the mean summation of all 3 tissue types’ values. Mean baseline levels of bncfDNA among healthy individuals are: total bncfDNA (mean 1.32 copies/ml, std 3.2), astrocytes cfDNA (mean 1.76, std 5.4), oligodendrocytes cfDNA (mean 0.5, std 2.7), neurons cfDNA (mean 0.9, std 2.9). BncfDNA: brain-derived circulating DNA.
