# Supplementary material for: Short report: Plasma based biomarkers detect radiation induced brain injury in cancer patients treated for brain metastasis: A pilot study
Source: PLoS One. 2023 Nov 28;18(11):e0285646. doi: 10.1371/journal.pone.0285646 (PMC10684068; doi:10.1371/journal.pone.0285646)
Supplement: S8 Fig — BncfDNA levels in undetermined radiological progression (URP) following brain radiotherapy. Documentation of URP in 4 patients is marked in a brown arrowhead. Each colored line represents a specific tissue origin of bncfDNA as detailed in the key (astrocytes, neurons, oligodendrocytes). Total bncfDNA marked in purple represent the mean summation of all 3 tissue type values. Mean baseline levels of bncfDNA among healthy individuals are: total bncfDNA (mean 1.32 copies/ml, std 3.2), astrocytes cfDNA (mean 1.76, std 5.4), oligodendrocytes cfDNA (mean 0.5, std 2.7), neurons cfDNA (mean 0.9, std 2.9). BncfDNA: brain-derived circulating DNA. (DOCX) [file pone.0285646.s008.docx]

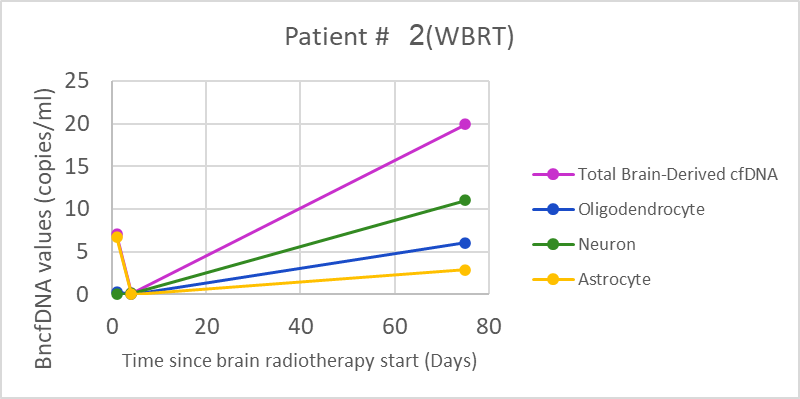


BncfDNA values (copies/ml)

Patient # 4 (WBRT)

Patient # 2 (WBRT)


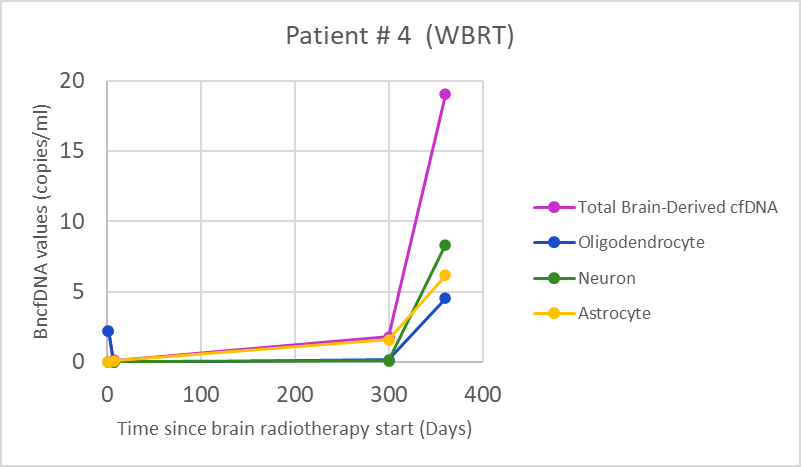


BncfDNA values (copies/ml)

Time since brain radiotherapy start (Days) y start (Days)

Time since brain radiotherapy start (Days) y start (Days)

Patient # 19 (WBRT)

Patient # 8 (WBRT)


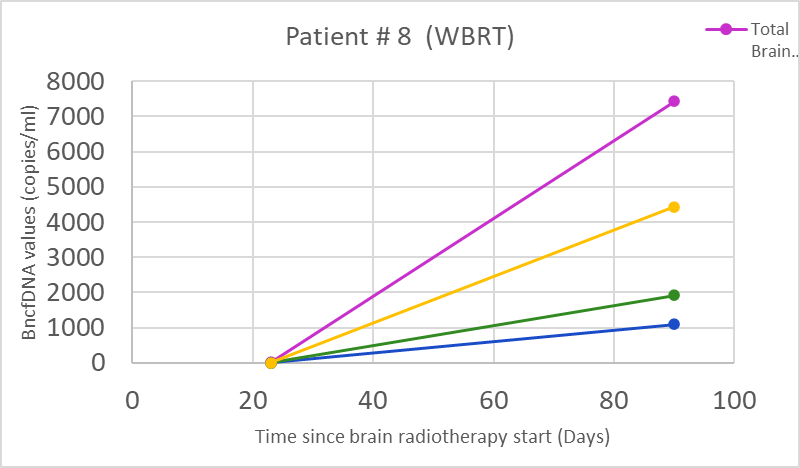

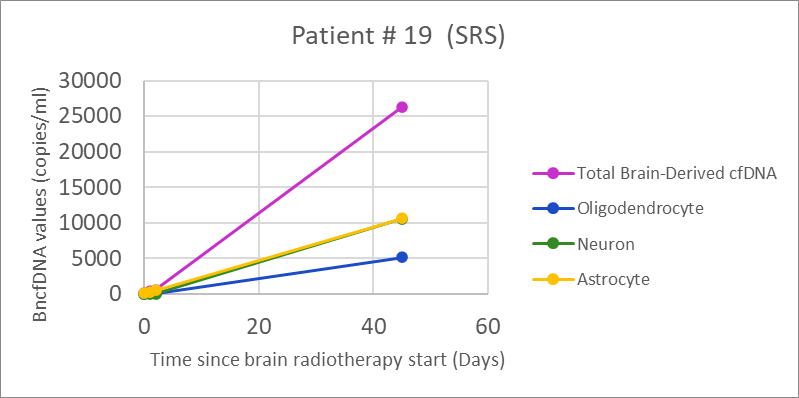


BncfDNA values (copies/ml)

BncfDNA values (copies/ml)

Time since brain radiotherapy start (Days) y start (Days)

Time since brain radiotherapy start (Days) y start (Days)

Time since brain radiotherapy start (Days) y start (Days)


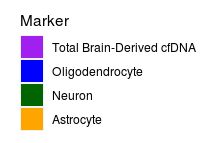


Total brain derived cfDNA

Oligodendrocyte derived cfDNA

Neuron derived cfDNA

Astrocyte derived cfDNA

URP

**Figure S8**: BncfDNA levels in undetermined radiological progression (URP) following brain radiotherapy. Documentation of URP in 4 patients is marked in a brown arrowhead. Each colored line represents a specific tissue origin of bncfDNA as detailed in the key (astrocytes, neurons, oligodendrocytes). Total bncfDNA marked in purple represent the mean summation of all 3 tissue type values. Mean baseline levels of bncfDNA among healthy individuals are: total bncfDNA (mean 1.32 copies/ml, std 3.2), astrocytes cfDNA (mean 1.76, std 5.4), oligodendrocytes cfDNA (mean 0.5, std 2.7), neurons cfDNA (mean 0.9, std 2.9). BncfDNA: brain-derived circulating DNA.
