# Supplementary material for: Short report: Plasma based biomarkers detect radiation induced brain injury in cancer patients treated for brain metastasis: A pilot study
Source: PLoS One. 2023 Nov 28;18(11):e0285646. doi: 10.1371/journal.pone.0285646 (PMC10684068; doi:10.1371/journal.pone.0285646)
Supplement: S9 Fig — Imaging studies reflecting radiotherapy effects. MRI of patient 7 before and 3 months after WBRT (A) showing decrease of enhancing lesions on T1GAD (bottom, arrowhead), accompanied with increase in periventricular white matter changes on FLAIR (top, white arrow), reflecting early delayed RBI. MRI of patient 22 before SRS and 2 and 4 months later (B) showing progressive increase in enhancing lesion on TaGAD (top., white arrow), compatible with radiation effects reflected by red signal on TRAM (bottom, black arrow), thus reflecting early-delayed RBI. WBRT: whole brain radiotherapy; SRS: stereotactic radiosurgery. (DOCX) [file pone.0285646.s009.docx]

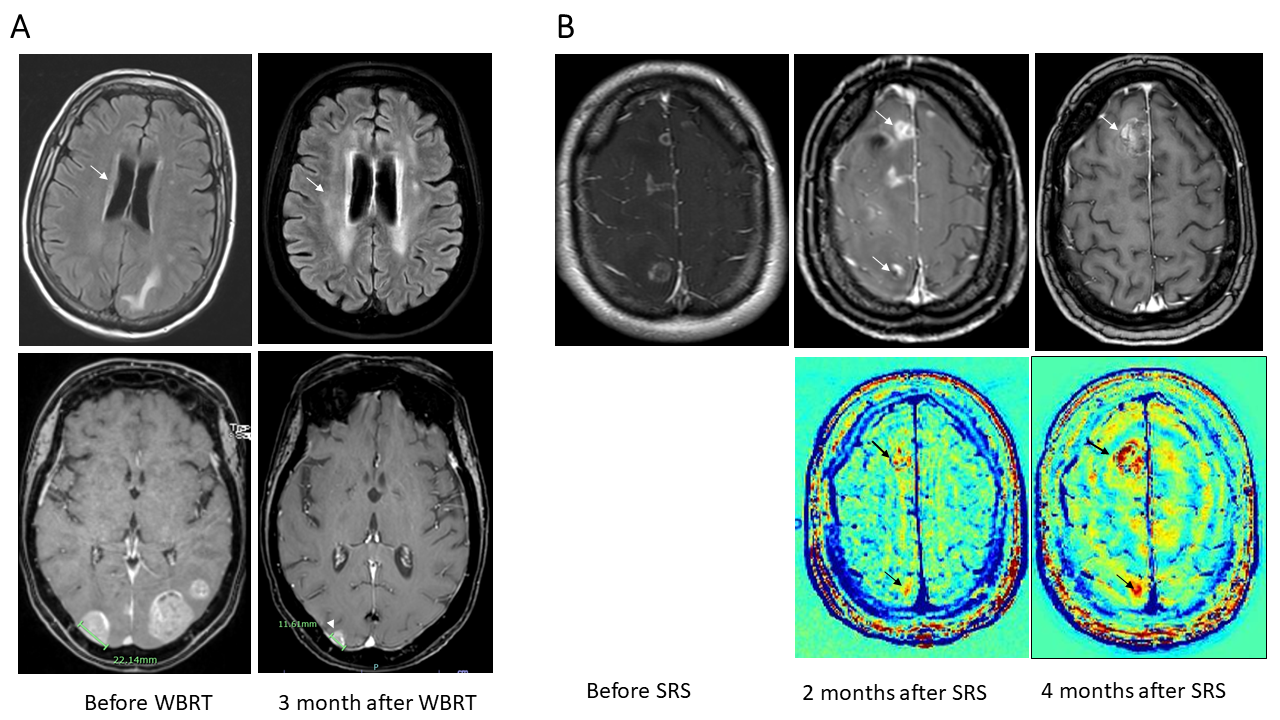


**Figure S9:** Imaging studies reflecting radiotherapy effects. MRI of patient 7 before and 3 months after WBRT (**A**) showing decrease of enhancing lesions on T1GAD (bottom, arrowhead), accompanied with increase in periventricular white matter changes on FLAIR (top, white arrow), reflecting early delayed RBI. MRI of patient 22 before SRS and 2 and 4 months later (**B**) showing progressive increase in enhancing lesion on TaGAD (top., white arrow), compatible with radiation effects reflected by red signal on TRAM (bottom, black arrow), thus reflecting early-delayed RBI. WBRT: whole brain radiotherapy; SRS: stereotactic radiosurgery.
