# Supplementary material for: Short report: Plasma based biomarkers detect radiation induced brain injury in cancer patients treated for brain metastasis: A pilot study
Source: PLoS One. 2023 Nov 28;18(11):e0285646. doi: 10.1371/journal.pone.0285646 (PMC10684068; doi:10.1371/journal.pone.0285646)
Supplement: S10 Fig — Ratio of total BncfDNA to total cfDNA levels measured for each patient during follow-up period, grouped to patients with RBI (blue lines) and non-RBI (red lines). Specific time points in which RBI and non-RBI occurred are marked as detailed in the legend (RIBD- blue star; treatment response–red hexagon; stable disease–red rhomboid; progressive disease–red square). (DOCX) [file pone.0285646.s010.docx]

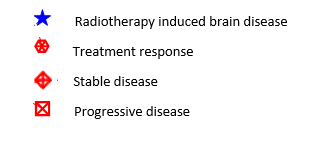


Tumor response

**Figure S10**: **BncfDNA ratio dynamic during RBI and non-RBI.** Ratio of total BncfDNA to total cfDNA levels measured for each patient during follow-up period, grouped to patients with RBI (blue lines) and non-RBI (red lines). Specific time points in which RBI and non-RBI occurred are marked as detailed in the legend (RIBD- blue star; treatment response – red hexagon; stable disease – red rhomboid; progressive disease – red square).
