# Supplementary material for: Short report: Plasma based biomarkers detect radiation induced brain injury in cancer patients treated for brain metastasis: A pilot study
Source: PLoS One. 2023 Nov 28;18(11):e0285646. doi: 10.1371/journal.pone.0285646 (PMC10684068; doi:10.1371/journal.pone.0285646)
Supplement: S1 Table — Each case of radiation-induced brain injury (RBI) is detailed by the patient in which it occurred (patient #), the day after radiotherapy initiation in which it presented (presenting day), the radiotherapy modality, the clinical manifestation and imaging presentation correlated to the time of RBI occurrence. Abbreviation: WBRT, whole brain radiotherapy; SRS, stereotactic radiosurgery; TRAM, treatment response assessment map; NA, non-available. (DOCX) [file pone.0285646.s011.docx]

**Table S1: Individual clinical details of RBI cases**

| **Definition** | **Patient #** | **Presenting day** | **Radiotherapy Modality** | **Clinical Manifestation** | **Imaging Presentation** |
| --- | --- | --- | --- | --- | --- |
| Acute | 1 | 4 | WBRT | Cerebellar signs and somnolence | NA |
| Acute | 10 | 9 | WBRT | Weakness and inability to walk | NA |
| Acute | 11 | 7 | WBRT | Central facial palsy | Stable lesion on brain CT |
| Acute | 14 | 3 | WBRT | Dizziness and visual disturbances | NA |
| Early delayed | 7 | 90 | WBRT | Ataxia | MRI – Periventricular white matter changes, no progression of BM |
| Early delayed | 22 | 60 | SRS | Stable | TRAM – lesion enlargement related to radiotherapy induced effects |
| Early delayed | 24 | 60 | SRS | Stable | TRAM – lesion enlargement related to radiotherapy induced effects |
| Late delayed | 4 | 300 | WBRT | Short-term memory deficit | MRI - Reduced edema and lesions size with periventricular white matter changes |
| Late  delayed | 18 | 240 | SRS | Headache and hemiparesis | TRAM – lesion enlargement related to radiotherapy induced effects |

**Table S1. Individual clinical details of RBI events.** Each case of radiation-induced brain injury (RBI) is detailed by the patient in which it occurred (patient #), the day after radiotherapy initiation in which it presented (presenting day), the radiotherapy modality, the clinical manifestation and imaging presentation correlated to the time of RBI occurrence.

*Abbreviation*: WBRT, whole brain radiotherapy; SRS, stereotactic radiosurgery; TRAM, treatment response assessment map; NA, non-available.
