# Supplementary material for: Short report: Plasma based biomarkers detect radiation induced brain injury in cancer patients treated for brain metastasis: A pilot study
Source: PLoS One. 2023 Nov 28;18(11):e0285646. doi: 10.1371/journal.pone.0285646 (PMC10684068; doi:10.1371/journal.pone.0285646)
Supplement: S2 Table — Each case of tumor status not related to RBI (response, progressive, stable) is detailed by the patient in which it occurred (patient #), the day after radiotherapy initiation in which it presented (presenting day), the radiotherapy modality, the clinical manifestation and imaging presentation correlated to the time of RBI occurrence. Cases of radiological progression which could not be solely attributed to RBI to tumor progression (URP) are also detailed as above. Abbreviation: RBI, radiation induced brain injury; PD, progressive disease; TR, tumor response; SD, stable disease; URP, undetermined radiological progression; LM, leptomeningeal; WBRT, whole brain radiotherapy; SRS, stereotactic radiosurgery; CSI, craniospinal irradiation; NA, non-available. (DOCX) [file pone.0285646.s012.docx]

**Table S2 Individual clinical details of tumor response**

| **Definition** | **Patient #** | **Presenting day** | **Radiotherapy Modality** | **Clinical Manifestation** | **Imaging Presentation** |
| --- | --- | --- | --- | --- | --- |
| Progressive Disease (PD) | 6 | 330 | WBRT (for LM) | Leg weakness | MRI - New brain metastases |
| Progressive Disease (PD) | 22 | 270 | SRS | Hand dysesthesia | MRI - New brain metastases on MRI and a new cervical spine lesion involving C7-T1 spinal root. |
| Progressive Disease (PD) | 23 | 270 | SRS | Cerebellar symptoms | MRI - New brain metastases |
| Undetermined radiologic progression (URP) | 2 | 45 | WBRT (for LM) | Seizure and coma | CT - Progressive hydrocephalus |
| Undetermined radiologic progression (URP) | 4 | 365 | WBRT | Stable | MRI - Progressive white matter changes and mixed tumor response |
| Undetermined radiologic progression (URP) | 8 | 90 | WBRT (CSI for LM) | Encephalopathy | MRI - Progressive hydrocephalus  (Spinal MRI – improvement after CSI) |
| Undetermined radiologic progression (URP) | 19 | 45 | SRS | Hemi-hypoesthesia | MRI - Enlargement of treated metastasis in addition to new brain metastases on MRI |
| Tumor Response (TR) | 4 | 7 | WBRT | Asymptomatic (resolution of dysarthria) | NA |
| Tumor Response (TR) | 12 | 6 | WBRT | Improvement in nausea and vomiting | NA |
| Tumor Response (TR) | 16 | 9 | WBRT | Improvement in dysarthria and dysphagia | NA |
| Tumor Response (TR) | 5 | 65 | WBRT | Improvement of dysphasia | MRI - Reduced lesion size |
| Tumor Response (TR) | 17 | 45 | SRS | NA | MRI - Complete response: no evidence for BM |
| Tumor Response (TR) | 18 | 90 | SRS | Asymptomatic | MRI - Partial response: reduction of lesion size |
| Tumor Response (TR) | 20 | 45 | SRS | Improvement of headache and seizures | MRI - Partial response: reduction of lesion size |
| Stable Disease (SD) | 3 | 8 | WBRT | Short term memory impairment | NA |
| Stable Disease (SD) | 9 | 4 | WBRT | Paraparesis, dysphagia, trigeminal hypoesthesia | NA |
| Stable Disease (SD) | 15 | 4,6 | WBRT | Ptosis | NA |
| Stable Disease (SD) | 18 | 26 | SRS | Asymptomatic | NA |
| Stable Disease (SD) | 22 | 21 | SRS | Asymptomatic | NA |

**Table S2**. **Individual clinical details of events related to non-RBI tumor status**. Each case of tumor status not related to RBI (response, progressive, stable) is detailed by the patient in which it occurred (patient #), the day after radiotherapy initiation in which it presented (presenting day), the radiotherapy modality, the clinical manifestation and imaging presentation correlated to the time of RBI occurrence. Cases of radiological progression which could not be solely attributed to RBI to tumor progression (URP) are also detailed as above.

*Abbreviation****:*** RBI, radiation induced brain injury; PD, progressive disease; TR, tumor response; SD, stable disease ; URP, undetermined radiological progression ; LM, leptomeningeal; WBRT, whole brain radiotherapy; SRS, stereotactic radiosurgery; CSI, craniospinal irradiation; NA, non-available.
