# Supplementary material for: Short report: Plasma based biomarkers detect radiation induced brain injury in cancer patients treated for brain metastasis: A pilot study
Source: PLoS One. 2023 Nov 28;18(11):e0285646. doi: 10.1371/journal.pone.0285646 (PMC10684068; doi:10.1371/journal.pone.0285646)
Supplement: S3 Table — Clinical, Radiological, and temporal criteria for definition and classification of radiotherapy-induced brain injury. Abbreviation: TRAM, treatment response assessment map. (DOCX) [file pone.0285646.s013.docx]

**Table S3 Criteria for RBI definition and classification**

| Type | Onset after start of radiotherapy | Neurological symptoms | Imaging findings |
| --- | --- | --- | --- |
| Acute | 1-30 days | One or more of the above:   - New/worsening headache - Cognitive impairment (effecting memory, orientation, executive function) - Ataxia - Focal neurological deficit (cranial neuropathy, motor, sensory, cerebellar, speech) - Intracranial pressure - New seizures | Not mandatory.  Can present peritumoral/ brain/edema |
| Early delayed | 1-6 months | Either criteria (1) or (2):   1. Asymptomatic with appropriate imaging finding 2. One or more of the above:  - New/worsening headache - Cognitive impairment (effecting memory, orientation, executive function) - Ataxia - Focal neurological deficit (cranial neuropathy, motor, sensory, cerebellar, speech) - Intracranial pressure - New seizures | One or more of the above must appear:   1. Enlargement of treated lesion with TRAM study confirming treatment response and not tumor progression 2. Periventricular encephalopathy or new white matter changes |
| Late delayed | >6 months | Either criteria (1) or (2):   1. Asymptomatic with appropriate imaging finding 2. One or more of the above:  - New/worsening headache - Cognitive impairment (effecting memory, orientation, executive function) - Ataxia - Focal neurological deficit (cranial neuropathy, motor, sensory, cerebellar, speech) - Intracranial pressure - New seizures | One or more of the above:   1. Enlargement of treated lesion with TRAM study confirming treatment response and not tumor progression 2. Periventricular encephalopathy or new white matter changes 3. New or progressing atrophy in brain area exposed to radiotherapy |

**Table S3. Criteria for RBI definition and classification.** Clinical, Radiological, and temporal criteria for definition and classification of radiotherapy-induced brain injury.

*Abbreviation*: TRAM, treatment response assessment map.
