# Supplementary material for: Short report: Plasma based biomarkers detect radiation induced brain injury in cancer patients treated for brain metastasis: A pilot study
Source: PLoS One. 2023 Nov 28;18(11):e0285646. doi: 10.1371/journal.pone.0285646 (PMC10684068; doi:10.1371/journal.pone.0285646)
Supplement: S4 Table — Clinical and radiological criteria for definition and classification of non radiotherapy-induced brain injury (non RBI). Abbreviation: TRAM, treatment response assessment map. (DOCX) [file pone.0285646.s014.docx]

**Table S4 Criteria for non-RBI definition and classification**

| Type | Neurological symptoms | Imaging findings |
| --- | --- | --- |
| Progressive Disease (PD) | Either criteria (1) or (2):   1. Asymptomatic with appropriate imaging finding 2. One or more of the above:  - New/worsening headache - Cognitive impairment (effecting memory, orientation, executive function) - Ataxia - Focal neurological deficit (cranial neuropathy, motor, sensory, cerebellar, speech) - Intracranial pressure - New seizures | 1. New or worsening metastases with clinical deterioration   AND   1. No evidence for radiation-induced changes appears in white matter or evident by TRAM imaging |
| Tumor Response (TR) | Improvement of neurological symptoms | Decrease in treated lesion size |
| Stable Disease (SD) | No change in neurological presentation | 1. No change in treated lesion size   AND   1. No new lesions   AND   1. No evidence for radiation-induced changes appears in white matter or evident by TRAM imaging |

**Table S4. Criteria for non-RBI definition and classification.** Clinical and radiological criteria for definition and classification of non radiotherapy-induced brain injury (non RBI).

*Abbreviation*: TRAM, treatment response assessment map.
