# Supplementary material for: Short report: Plasma based biomarkers detect radiation induced brain injury in cancer patients treated for brain metastasis: A pilot study
Source: PLoS One. 2023 Nov 28;18(11):e0285646. doi: 10.1371/journal.pone.0285646 (PMC10684068; doi:10.1371/journal.pone.0285646)
Supplement: Attachment — Submitted filename: Responses to Reviewrs (final) (1).docx [file pone.0285646.s017.docx]

Response to Reviewers:

Dear Reviewers,

Thank you for reviewing our manuscript. We appreciate your comments and helpful suggestions.

We revised our paper according to reviewers` suggestions. First, we revised our terminology to the term “radiation-induced brain injury” as suggested. Also, following your advice, we edited Figure 1A to 2 separate figures (1A and 1B). Importantly we also reviewed and corrected the manuscript for grammar and syntax as recommended.

These changes and others are summarized below (marked in red color below each comment). line numbers (marked in blue color) refer to the version of “revised manuscript highlighted with track changes”.

Reviewer #1: The authors have responded thoroughly to the reviews. The work remains intriguing and novel. There are a few issues that remain that need to be addressed prior to acceptance for publication.

1. Figure 1A looks quite busy and the pattern / association with RIN/RIBD is not clear and does not support the idea that increases in cfDNA tightly correlate with brain injury. However, this may be due to noise and variation inherent to the data. Perhaps two figures would be better. One panel could look only at XRT patients without RIBD (red lines), anchored by start of XRT (align all the curves with respect to clinical onset of RIBD instead of XRT start). The RIBD panel (blue lines) might be better anchored by the date of RIBD. The patterns are just quite difficult to see in the figure 1A, which looks quite noisy, and look like they argue against any real pattern, but I suspect this can be teased out using an approach something like the above.
Adding the detailed individual case courses is very helpful. Thank you for reporting these data.

Thank you for these helpful suggestions. We edited Figure 1A as recommended, by splitting it to 2 parts: 1A (to represent patients with RIBD) and 1B (to represent patients non-RBI) and highlighting clinical onset of each event. We hope it is clearer now for visualization and interpretation.

2. For patient #11 WBRT – they appeared to have a single spike in cfDNA – did their symptoms only last a few days and then resolve? Or did they persist after cfDNA levels returned to normal? This is worth a brief comment/discussion.

I will refer my response to patient #14 that indeed has only 2 time-points of cfDNA measurement instead of patient #11 (that have 9 measurement time-points). Following one day after clinical onset his neurological symptoms were stable. Unfortunately, we were not able to continue following patient #14 later, since he was transferred to other hospital for palliative care due to systemic progression of his cancer, from which he deceased 35 days later.

3. The revisiting of the terminology is appreciated. However, in the cited paper the term “Radiation-Induced Brain Injury” appears to be used far more often than “Radiation-Induced Brain Disease” (17 vs. 3 times). The term “disease” implies a more sustained, potentially permanent condition and physiologic entity, whereas the term “Radiation Inducted Brain Injury” would be perfect and corresponds nicely to what the authors are directly claiming to measure – injury of brain cells (both neurons and glia). The term “Radiation-Induced Brain Injury” should be used throughout to avoid confusion or a pejorative connotation.

We changed the term “Radiation-induced brain disease” to the term “Radiation-Induced Brain Injury” in the manuscript, as well as in the title and in all associated figures and tables.

Minor comments:
1. **Table 1** is quite simplistic and these data are probably better just described in the text, separating the descriptions of the SRS vs. WBRT patients with respect to timing of RIBD events (these are very different clinical contexts).

As suggested, we omitted table 1 and described the important patient’s characteristic in the text on the results section (lines 137-140).

2. Table S1: The definition column might be more clear to just state “Acute, Early-Delayed, or Late Delayed” instead of acronyms like ARIBD, EDRIBD, LDRBID.

We corrected this column as suggested.

3. Table S2: Can an alternative to “URP” like “PD” be use for progressive disease?

In this work we use the term “progressive disease” to reflect tumor progression, in contrast to cases of radiological progression resulting from radiation effects on the peritumoral tissue such as peritumoral edema and inflammatory response (here referee as “Radiation induced brain injury”). We used the term
“undetermined radiological progression (URP)” in cases that we could not attribute the radiological progression specifically to either tumor enlargement (“PD”) or peritumoral enlargement (“RBI”), In order to differentiate between those 2 entities. Since we cannot say clearly that those cases defined as “URP” are related to tumor progression we would avoid the use of the term “progressive disease” in those specific undetermined cases.

4.Table S4: Does the X axis indicate time? Is this to scale? If so time should be added. The figure is helpful for spelling out the clinical course of each patient.

Thank you for this comment. Assuming this important comment refers to figure S1 (right after table S4) - We added in figure S1 a label for the X axis which indicates it relates to time.

Reviewer #3: The authors are to be applauded for seeking to identify biomarkers for treatment-related neurotoxicity in patients with brain metastases using peripheral blood methylation markers previously described. The limitations of the article were appropriately recognized by the previous reviewers and the authors have made attempts to rectify the manuscript appropriately. The decision to convert to a short report and to present the study findings as hypothesis generating is appropriate, given the small sample size and other previously specified limitations.

1.The authors should consider clarifying the eligibility criteria for patients included in this study.

Thank you for this comment. As suggested, we elaborate eligibility criteria in the “Methods” section describing patient population (lines 72-74).

2.The authors should also review the manuscript for clarity and **English grammar** and should consider limiting the extensive use of **acronyms** in the manuscript text.

We reviewed the manuscripts and rephrased several sentences to be clearer, as well as several corrections related to grammar and syntax. As suggested we also omitted some of the abbreviations and used instead its full terms (“brain metastases”, “early-delated”, “ late-delayed”, progressive disease”, “tumor response”, “stable disease”). ( lines: 24, 25, 28, 30, 37, 44, 55, 62, 63, 67-68, 72, 73-74, 85-87, 92, 98-104, 123, 173-176, 181, 184187, 189, 191, 193, 195, 199-200, 217, 222, 225, 228, 230, 231, 233, 236, 238, 242, 244, 248, 250, 264, 265, 270, 272, 274, 275),

3. Finally, the authors should specify which cases of tumor progression or radionecrosis were biopsy confirmed, and acknowledge the limitations of interpreting findings without a biopsy.

We added a sentence to reflect this limitation in lines 88-89, and in the discussion section, in lines 281-282.
